# An Integrated Approach to Uncover Driver Genes in Breast Cancer Methylation Genomes

**DOI:** 10.1371/journal.pone.0061214

**Published:** 2013-04-08

**Authors:** Xiaopei Shen, Shan Li, Lin Zhang, Hongdong Li, Guini Hong, XianXiao Zhou, Tingting Zheng, Wenjing Zhang, Chunxiang Hao, Tongwei Shi, Chunyang Liu, Zheng Guo

**Affiliations:** 1 Bioinformatics Centre, School of Life Science, University of Electronic Science and Technology of China, Chengdu, China; 2 Department of Bioinformatics, School of Basic Medical Sciences, Fujian Medical University, Fuzhou, China; Michigan State University, United States of America

## Abstract

**Background:**

Cancer cells typically exhibit large-scale aberrant methylation of gene promoters. Some of the genes with promoter methylation alterations play “driver” roles in tumorigenesis, whereas others are only “passengers”.

**Results:**

Based on the assumption that promoter methylation alteration of a driver gene may lead to expression alternation of a set of genes associated with cancer pathways, we developed a computational framework for integrating promoter methylation and gene expression data to identify driver methylation aberrations of cancer. Applying this approach to breast cancer data, we identified many novel cancer driver genes and found that some of the identified driver genes were subtype-specific for basal-like, luminal-A and HER2+ subtypes of breast cancer.

**Conclusion:**

The proposed framework proved effective in identifying cancer driver genes from genome-wide gene methylation and expression data of cancer. These results may provide new molecular targets for potential targeted and selective epigenetic therapy.

## Introduction

Abnormality in DNA methylation plays an important role in cancer initiation and progression. For example, it has been found that promoter hypermethylation of the *APC* (adenomatous polyposis coli) gene could increase *β-catenin* levels and lead to the activation of growth-promoting genes in colon and gastrointestinal cancer [1] and promoter hypomethylation of *Wnt5a* could increase this gene's transcriptional level to promote the aggressiveness of prostate cancer [2]. With the development of methylation microarray technology, thousands of gene promoters have been found to be either hyper- or hypomethylated in cancer genomes [3], [4]. However, only a small portion of these genes play “driver” roles in cancer initiation and progression, while the others are only “passengers” in the tumorigenic process [5], [6]. It is difficult to discriminate the drivers from the passengers [6] in a large number of genes differentially methylated in human cancer genomes [4], and the identification of driver genes with methylation alterations is a fundamental step towards molecular characterization of cancer.

Recently, using genome-wide methylation data, De Carvalho developed an approach to identify a specific type of driver genes for the survival of cancer cells [5]. However, a major limitation of this approach is that it can only capture driver genes with promoter hypermethylation. There are evidences that promoter hypomethylation of some genes may also be associated with the initiation and progression of cancer by regulating the activity of the genes [7]–[9].

Similar to copy number alteration, methylation alteration at gene promoters typically does not alter the coding sequences of genes, but contributes to cancer by influencing gene expression [10]. Previous research has defined driver copy number alterations based on the assumption that a driver gene is expected to influence the expression of this gene and a group of downstream genes which affect particular cancer phenotypes [11], [12]. This assumption could also be applied to identify driver genes from methylation data. Considering the diversity of cancer phenotypes, we could modify the assumption to be that the downstream genes of a driver gene can affect cancer-associated pathways to induce corresponding cancer phenotypes [13].

Based on above assumption, we propose an approach to identify cancer driver genes using gene methylation and expression data of cancer. We applied this approach to analyze data for breast cancer to derive driver genes. Then, we provide evidence to validate these findings based on their links with known cancer genes on the protein-protein network. Finally, we further explore the subtype specificity of the identified driver genes of breast cancer.

## Materials and Methods

### DNA methylation and gene expression data

Three breast datasets with both methylation and expression data from Gene Expression Omnibus (GEO) [14] and The Cancer Genome Atlas (TCGA) (http://tcga-data.nci.nih.gov/tcga) were collected (Table 1). The gene promoter methylation data of Bre100 and Bre95 were collected with the Illumina HumanMethylation27 platform, which detected the methylation level of 27,578 CpG loci located within the proximal promoter regions of transcription start sites of 14,495 genes. The methylation data of Bre60 were collected with Illumina HumanMethylation450 platform, which detected the methylation level of over 450,000 CpG loci covering all gene regions, including the promoter and gene body. For Bre60, we extracted the loci at the promoter which overlapped that in the HumanMethylation27 for analysis. Using methylated signal intensity (M) and unmethylated signal intensity (U), the methylation level (beta-value) for each CpG locus was calculated by max (M, 0)/(|U|+|M|+100) [15]. We removed unreliable probes whose proportion of detection P-value>0.05 across all the samples was more than 10%. The 1,092 CpG loci within promoters of 605 sex chromosome genes were excluded from the analysis to eliminate gender-specific bias.

**Table 1:** The methylation data analyzed in this study.

Data	Sample size (cancer vs. normal)	Data source
Bre100	88:12	GSE:20713 [18]
Bre95	88:7	TCGA batch 85
Bre60	46:14	TCGA batch 61

For the samples of Bre100, gene expression was available simultaneously using Affymetrix Human Genome U133 Plus 2.0 Array. The raw gene expression profiles were normalized using the robust multi-array analysis (RMA) algorithm [16]. The probe IDs were mapped to Gene IDs with the annotation table for each platform. The expression data of Bre95 and Bre60 were collected with the normalized data of Agilent4502A platform. Using a T-test, genes with adjusted P values less than 0.05 were defined as differentially expressed (DE) genes [17].

The subtyping of cancer samples in Bre100 was determined according to the expression of estrogen receptor (ER) and human epidermal growth factor receptor 2 (Her2) by immunohistochemistry (IHC) [18].

### Cancer genes and protein-protein interaction (PPI) data

We extracted 2104 cancer genes from the Cancer Gene F-Census [19] which is a collection of cancer genes from various data sources such as the Cancer Gene Census database [20] and the Tumor Suppressor Gene database [21].

The human PPI data was downloaded from MINT [22], BIND [23], IntAct [24], HPRD [25], MIPS [26], DIP [27], KEGG (Kyoto Encyclopedia of Genes and Genomes) (PPrel for PPI and ECrel for enzymes involved in neighboring steps) [28] and Reactome protein pairs involved in a complex and neighboring reaction [29]. The types of pair-wise relationships between proteins include “interact with”, “metabolic catalysis”, “component of”, “co-control” and “sequential catalysis”. For simplicity, we used the term “interaction” to represent various relationships between proteins and designated this network as the protein interaction network. We pooled together the eight PPI datasets [30] and compiled an integrated PPI network of 142,583 distinct interactions involving 13,693 human proteins.

### Discretization of methylation profiles for individual cancer samples

**Figure 1:**
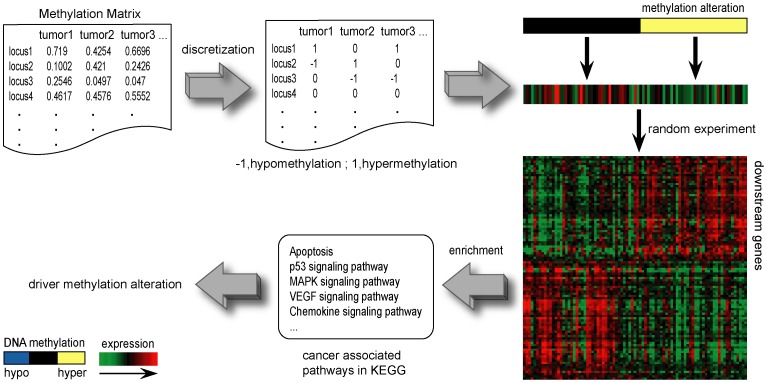
Schematic overview of the approach. Methylation matrix of continuous beta values is transformed into a discrete profile by comparing with the methylation profiles of normal samples by discretization (1 denotes hypermethylation, −1 denotes hypomethylation and 0 denotes no differential methylation). Identification of driver alteration required following three conditions. Firstly, for each locus, if its gene expression was significantly down- or up-regulated in hyper- or hypomethylated cancer samples comparing with the cancer samples which had no differential methylation at this locus (T-test, FDR<0.05), it is retained for follow analysis. We showed the hypermethylated locus (labeled with yellow) as an example. Secondly, the methylation alterations which influence the expression of significantly more downstream genes were selected (see Methods). Thirdly, downstream genes of a driver methylation alteration should be enriched in at least one of the cancer-associated pathways.

Data discretization was used to identify the state of differential methylation for a locus in a sample. We identified a locus that was hyper- or hypomethylated in each cancer sample by comparing the methylation value with those of the normal samples (Figure 1). Specifically, we normalized the methylation values of the locus in cancer samples as a Z-score, utilizing mean and standard deviation of methylation values of the locus in the normal samples [12]. A locus was considered differentially methylated if the normalized methylation value of the locus had an adjusted P-value<0.05 using a Z-test. Based on the sign symbol of Z-scores, the differentially methylated loci were classified into hypermethylated and hypomethylated ones. At last, the methylation profile of the cancer samples were translated into a matrix comprising of 1 (hypermethylation), 0 (no differential methylation) and −1 (hypomethylation).

### Identifying driver genes

According to the assumption mentioned in the *Introduction*, a locus with methylation alteration was identified as a driver, if it met the following three requirements.

Firstly, for each locus, we required that its gene expression was significantly down- or up-regulated in hyper- or hypomethylated cancer samples comparing with the cancer samples which had no differential methylation at this locus (T-test, false discovery rate (FDR)<0.05) [17] (Figure 1).

Secondly, a driver methylation alteration should influence the expression of downstream genes. The downstream genes were defined as the DE genes between tumor samples with this methylation alteration (hypermethylation of hypomethylation) and tumor samples with no differential methylation alteration. Random experiments were performed to see whether the number of downstream genes of the driver alteration was significantly more than expected by chance (FDR<1.00E-04). Specifically, we randomly extracted the same number of tumor samples as those with the methylation alteration and with no differential methylation, and subsequently performing the identification of DE genes for 100,000 times. The P-value of the observed number of DE genes was calculated as the percentage of the random numbers exceeding the observed number (Figure 1).

Thirdly, downstream genes of a driver methylation alteration should disturb at least one of the cancer-associated pathways (Figure 1). In relation to the disturbed cancer pathways, we selected 36 cancer-associated pathways (Table S1), by referring to the pathways annotated in “pathway in cancer” in KEGG [28] and the ten hallmarks of cancer [31]. The mapping of the pathways to cancer hallmarks was collected from previous reports [31]–[33].

If a methylation alteration meets the above three requirements, it was defined as a driver methylation alteration. A gene with at least one driver alteration locus was defined as a driver gene.

## Results

### Identification of driver genes for breast cancer

Different from mutation and copy number profiles, methylation profile is consisted of continuous methylation values, and it is hard to identify the differential methylation state of a CpG locus in each cancer sample. Thus, we preformed data discretization for methylation profiles of Bre100 at first. After data discretization, we restricted our analysis to 9029 methylation altered loci which were hyper- or hypomethylated in at least 10% of all cancer samples. If a gene was found both hyper- and hypomethylated in at least 10% cancer samples, it was excluded from follow analysis. Using the T-test with FDR<0.05, we identified 888 loci hypermethylated or hypomethylated within the promoters of 753 genes which were significantly down- or up-regulated in the cancer samples. From these 888 loci, we found 350 loci from 311 genes which influenced the expression alterations of significantly more downstream genes than expected by random chance according to the random experiments described in the *Methods*. Finally, from these 350 loci, we identified 249 loci of 222 genes whose downstream genes were significantly enriched in at least one of the cancer-associated pathways defined in Table-S1 (hypergeometric test, FDR<1.00E-04). (Table S2).

By the same procedure, we identified 189 and 58 driver genes in the Bre95 and Bre60 datasets, respectively (Table S2). The percentage of overlapping genes (POGs) between the list of driver genes extracted from Bre100 and the two lists of driver genes extracted from Bre95 and Bre60 were 12.25% and 26.10%, respectively, which were both significantly higher than that expected by random chance (hypergeometric test, P<1.11E-16). It should be recognized that each of the driver gene lists could only capture a portion of the effective biology signals associated with the tumorigenesis due to the lack of statistical power in most small-scale experiments [34], [35]. Thus, the three lists of the driver genes extracted from the three datasets were integrated for the following validation analysis.

### Validation of the identified driver genes

Pooling together the driver genes extracted from all three breast cancer datasets, we got 411 driver genes. Evidences supported that these driver genes are likely to play driver roles in tumorigenesis. Firstly, 82 (19.95%) of the identified 411 driver genes were known cancer genes collected in the F-census database [19], which was significantly more than expected by random chance (P = 1.07E-04) (Table 2). Specifically, the percentage of known cancer genes in the hypomethylated driver genes (19.66%) was also significantly higher than that expected by random chance (P = 1.18E-02), suggesting that these hypomethylated genes also played a driver role in tumorigenesis (Table 2). Secondly, in addition to the known cancer genes collected in the F-census database, many other driver genes have been suggested to be cancer genes in previous studies [36]–[38]. For instance, *PCDH8* has been identified as a driver gene with promoter hypermethylation, in accordance with a previous report that this gene might be a candidate tumor suppressor gene for breast cancer [37].

**Table 2:** The proportion of known cancer genes in our driver genes.

	Gene^#^	Genes in F-census^#^	P_F-census	Neighborson PPI^#^	P_PPI
Driver genes	411	82	1.07E-04	183	6.01E-04
Hypomethylated driver genes	178	35	1.18E-02	86	6.50E-03

After removing the 82 known cancer genes from the 411 identified driver genes, we found that the remaining 329 driver genes were significantly enriched in the direct interaction neighbors of known cancer genes collected in F-census (hypergeometric test, P = 6.01E-04). This result implied that many of the newly predicted driver genes worked closely with the known cancer genes and might perform similar functions as their neighboring cancer genes in tumorigenesis. For instance, it has been reported that cancer gene *TSG101* could perturb the cell cycle pathway in breast cancer [39]. In our analysis, its neighbouring gene *RRM2* was identified as a hypomethylated driver gene with its downstream genes disturbing cell cycle pathway, which also corresponded with previous finding that *RRM2* could disturb cell cycle and contributed to tumorigenesis [40]. Specifically, we observed that the direct interaction neighbors of known cancer genes were also significantly enriched with the hypomethylated driver genes (P = 6.50E-03), which at present were not known as cancer genes.

### Subtype-specific driver genes of breast cancer

**Figure 2:**
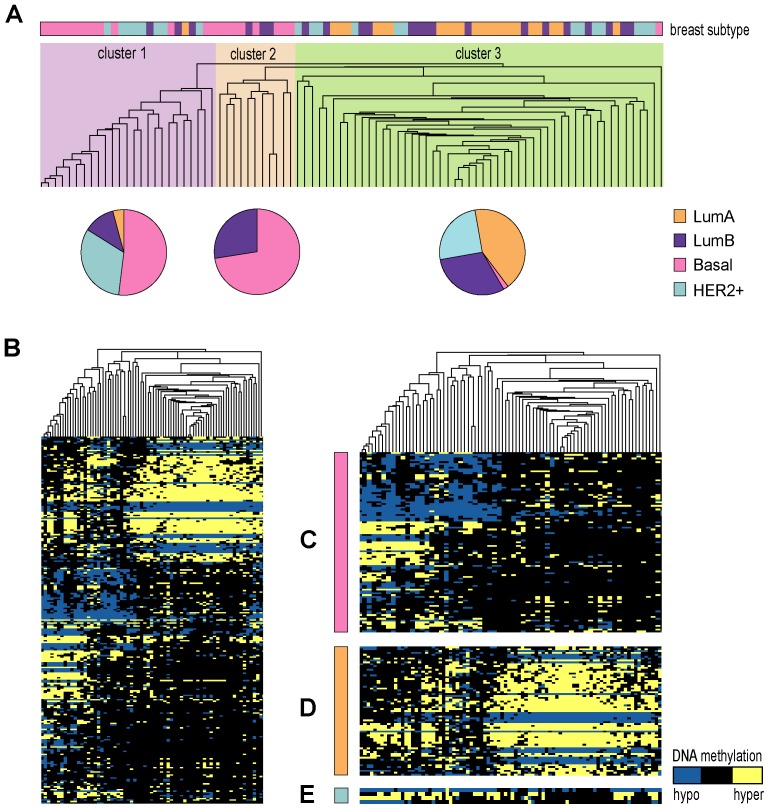
Hierarchical cluster analysis of the 88 tumor samples using discrete methylation profile of 222 driver genes. (**A**) Experimental dendrogram shows the clustering of the tumors into three subgroups: cluster 1(light purple, n = 25); cluster 2 (orange, n = 11); cluster 3 (light green, n = 52). The pie charts show the distribution of sample subtypes within each cluster. (B) Overview of complete cluster diagram. (C) Basal-like subtype-specific driver genes. (D) Luminal-A subtype-specific driver genes. (E) HER2+ subtype-specific driver genes.

Previous reports suggest that breast cancer has four subtypes with specific gene expression patterns [41], [42]. Therefore, we could assume that some subtype-specific expression patterns might be caused by the subtype-specific driver methylation alterations. For this study, analysis was only performed on the driver genes extracted from the Bre100 dataset, since available subtype information was limited to this dataset only. Based on unsupervised hierarchical clustering using the Jaccard correlation distance and average linkage [43] for the discretized methylation profiles of the 249 driver methylation alterations loci of the 222 driver genes in the Bre100 dataset, the 88 cancer samples were divided into three clusters (Figure 2-A, 2-B). We found that luminal-A samples were mostly in cluster 3, basal-like samples were mainly in clusters 1 and 2 and all HER2+ samples were in clusters 1 and 3, indicating that these three subtypes may have subtype-specific driver methylation alterations.

Using the hypergeometric test, we selected subtype-specific driver genes whose alterations occurred significantly more frequently in the samples of a particular subtypes than in samples of other subtypes. With FDR<0.05, we found 89 basal-like specific driver genes, 64 luminal-A specific driver genes and 4 HER2+ specific driver genes (Figure 2-C, 2-D, 2-E). For instance, *HDAC1* was identified as a basal-like specific driver gene as it displayed a significantly higher frequency of hypomethylation (63.64%) in basal-like tumors than in the other subtypes (25.76%) (P = 1.70E-03).

**Figure 3:**
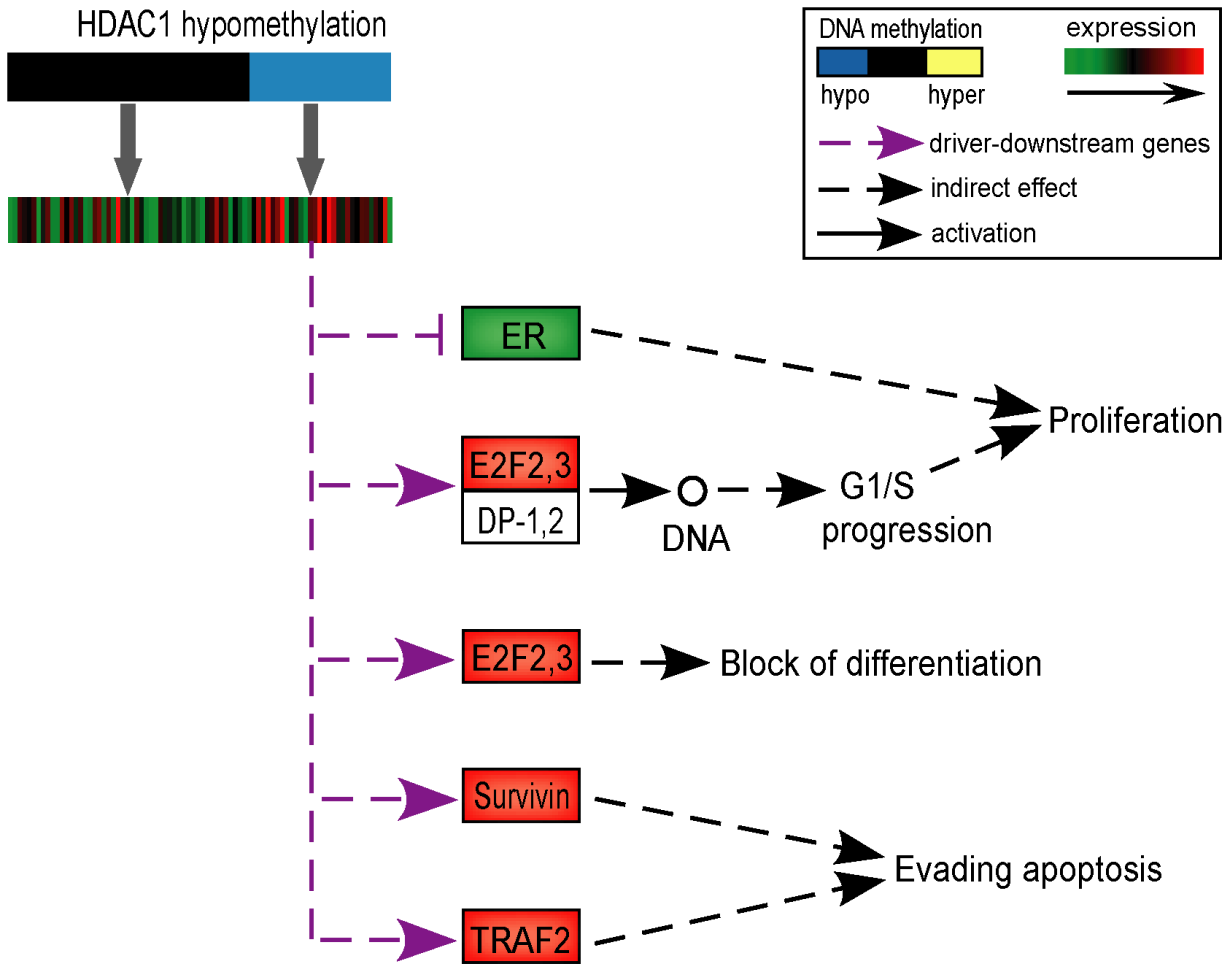
Downstream genes of hypomethylated HDAC1. The downstream genes with functional consequence in the KEGG “pathway in cancer” were selected. The purple arrows imply the relationship between driver gene and its downstream genes, and the dark arrows were collected from “pathway in cancer” of KEGG. The up-regulated genes are labeled with red color and the down-regulated genes are labeled with green color.

It has previously been reported that *HDAC1* could interact with ER-α to suppress ER-αtranscription activity [44] in accordance with the feature of the basal-like subtype that it is ER-negative samples. To further investigate the role of *HDAC1* in basal-like tumors, we mapped the downstream genes of *HDAC1* into the “pathway in cancer” of KEGG and found that the changes of their expressions could block the differentiation of cells, promote proliferation and evade apoptosis (Figure 3), which corresponds with a previous report about HDAC1 [45]. Specifically, the downstream genes *E2F-2,3* coordinate with *DP-1,2* and their up-regulation could promote the transcription of S-phase genes encoding for proteins that amplify the G1 to S-phase switch, which could speed up DNA replication and cell proliferation. Meanwhile, the up-regulation of *E2F-2,3* could also block the differentiation of cells. Similarly, the up-regulation of the downstream gene *TRAF2* could bind to cellular inhibitors of apoptosis for tumor necrosis factor (*TNF*) to efficiently activate *NF-κB* and prevent *TNF*-induced apoptosis [46]. This could explain why basal-like subtype samples usually have high proliferation and low differentiation rates [47], [48].

## Discussion

Although a large number of aberrant methylation alterations in cancer genomes have been found, it is still difficult to identify driver methylation alterations from them. Identification of the driver genes with methylation alterations and their downstream genes is a fundamental step towards the mechanistic characterization of cancer. Furthermore, this may provide new targets for potential targeted and selective epigenetic therapy considering the reversibility of methylation [49]. In this study, we proposed a computational approach to identify driver genes by taking into account not only the association between promoter methylation and gene expression, but also the association between a candidate driver and its downstream genes. Additionally, the pathways represented by the downstream genes can help us gain insight into how a driver methylation alteration contributes to the malignant phenotype through altering the cellular pathways. Notably, the enrichment of hypomethylated driver genes with known cancer genes provided evidence that hypomethylation of gene promoters are also closely linked to the initiation and progression of cancer. Because it is usually believed that global hypomethylation of DNA in cancer is closely associated with repeated DNA elements, methods in identifying genes with driver methylation alteration have usually focused on promoter hypermethylation [5], [50], and cancer-associated promoter hypomethylation receive relatively little attention [51]. In the present study, we have shown a procedure that makes it possible to not only capture the genes with driver hypermethylation, but also the genes with driver hypomethylation.

Using this procedure to analyze the data for breast cancer, we identified many driver genes with evidence that they were closely linked with known cancer genes on the protein-protein network. Specifically, the subtype-specific driver methylations suggested that methylation plays a significant role in differentiating breast tumor subtypes and might be potential targets for the subtype diagnosis and therapy. Evidence exists that the knockdown of HDAC1, which is a basal-like subtype-specific driver gene, could cause cell cycle arrest, growth inhibition and apoptosis in breast cancer cells [45]. It has also been shown that the inhibitor of HDAC1, panobinostat, is overtly toxic to the cells of basal-like samples, and causes a decrease in tumorigenesis *in vivo* [52].

An important step of our method is the discretization of continuous methylation profile for combining information at the level of individuals. It was shown to provide a way for integration analysis for the expression and methylation data. However, the selection of the threshold for identifying the alterations at individual level might influence the statistical power for determining the driver genes. Therefore, we have additionally performed our approach with discrete methylation profiles using another threshold of FDR<0.01 for identifying the alterations at individual level. This produced similar results that the predicted driver genes are still significantly enriched with known cancer genes (P = 3.75E-04). Another potential difficulty in our approach is that there is currently no official definition of cancer-associated pathways. The cancer-associated pathways that we selected mostly came from the cancer hallmark based on published literature [31]. As the definition of cancer-associated pathways is improved, the performance of our procedure would also improve. Notably, the potential oncogenic roles of the newly predicted driver genes based on computational analysis need to be confirmed by further wet bench experiments.

Finally, we note that except for methylation alteration, mutation, copy number change, microRNA change [53] and other epigenetic modifications such as histone modification [54] can also influence the expression of driver genes. Therefore, future studies are needed to integrate these types of molecular alterations and improve the method for identifying driver genes of cancer.

## Supporting Information

Table S1 Cancer-associated pathways. List of 37 cancer-associated pathways from KEGG and the literatures.
(XLS)

Table S2 Driver genes in three breast cancer datasets. List of 249, 205 and 62 driver methylation loci identified from Bre100, Bre95 and Bre60 datasets, respectively with the cancer-associated pathways significantly enriched with corresponding downstream genes (0, false;1, true).
(XLS)
